# Emerging trends in digital banking: technologies, usability, regulation, and transformation of the financial sector

**DOI:** 10.12688/f1000research.166554.1

**Published:** 2025-07-24

**Authors:** Ada Gallegos, Luisa Adriana Rodríguez Zavala, Alejandro Valencia-Arias, Jackeline Valencia

**Affiliations:** 1 Instituto de Investigación de Estudios de la Mujer, Universidad Ricardo Palma, Santiago de Surco, 15039, Peru; 2 Vicerrectoría de Investigación y postgrado, Universidad de los Lagos, 5290000, Chile; 3 Facultad de Ciencias Económicas y Administrativas, Instituto Tecnologico Metropolitano, Medellín, Antioquia, 50010, Colombia

**Keywords:** Digital banking, emerging technologies, financial inclusion, financial regulation, digital transformation

## Abstract

Digital banking has been identified as a significant catalyst for transformation within the global financial system, driven by technological advancements and evolving user demands. This paradigm shift has the potential to profoundly impact the relationship between institutions and their customers, necessitating the development of novel business models and regulatory frameworks. However, the field of applied research exhibits methodological and thematic dispersion, which limits comprehensive understanding by hindering the connection between technological developments, regulatory perspectives, and user experiences. This, in turn, restricts the identification of clear patterns and the formulation of coherent strategies. In the context of a dynamic environment characterised by constant innovation and evolving expectations, there is a necessity to unify knowledge through systematic syntheses that organise information and guide future research. This approach involves the rigorous and systematic application of analytical methods, such as those grounded in the PRISMA methodology, to address the intricacies inherent in the interplay between emerging technologies, functionalities, and the social and institutional challenges they encounter. This transformation of digital banking into a multidimensional phenomenon necessitates integrated approaches to anticipate risks, promote inclusion, and strengthen a resilient and sustainable financial system.

## Introduction

Digital banking has been identified as a pivotal element in the ongoing transformation of the global financial system. The evolution of this field commenced with the advent of electronic technologies, such as automated teller machines (ATMs) and telephone banking, and has since progressed to encompass mobile platforms and digital financial services. This change has been driven by the advancement of information and communications technologies and by user demand for faster, more tailored, and more accessible solutions. ^1^

In the contemporary context, characterised by the digitalisation of economic and social processes, digital banking signifies more than mere technical progress. This paradigm shift entails a transformation in the relationship between financial institutions and their customers. Digital tools have been demonstrated to expand access to financial services for excluded populations, thus rendering digital banking a pivotal factor in the pursuit of financial inclusion. Consequently, its implementation necessitates the redesign of business models, service channels, security schemes and regulatory compliance mechanisms. ^2, 3^

The prevailing paradigm of consumer behaviour, characterised by an emphasis on expediency, autonomy and personalisation in the digital domain, compels institutions to engage in perpetual innovation. Digital banking, as a consequence, responds to a functional need, but it also modifies the relationship between technology, regulation and social expectations. This phenomenon has direct implications for the stability, competitiveness, and sustainability of the financial system. ^4^

In recent years, the rapid expansion of digital banking has given rise to a substantial corpus of academic and technical studies addressing its various dimensions. The extant publications address a range of topics, including but not limited to applied technologies, developed functions, user experience, current regulations, and adoption challenges. ^5^ However, despite the increasing volume of research, a clear gap remains in the systematic organisation of the knowledge generated.

The extant literature on the subject is fragmented, with a plethora of methodological approaches, varied geographical contexts, distinct disciplinary interests, and heterogeneous analytical frameworks. This hinders a comprehensive understanding of the phenomenon and obstructs the formulation of a coherent vision of emerging trends in digital banking. ^6, 7^ This fragmentation is evidenced by the parallel existence of studies focused on specific technologies, such as artificial intelligence, blockchain, or mobile applications, and others that analyse user perspectives, without a common framework linking both types of contributions.

In a similar vein, research concerning the regulation of digital transformation in banking frequently lacks integration with analyses of platforms’ adoption and functional development. ^5^ Consequently, extant knowledge is descriptive yet lacks the capacity to provide a comprehensive explanation of the dynamics that are shaping the contemporary digital banking environment. The digital financial sector is undergoing a period of rapid transformation, characterised by incessant innovation, intensified competition, and rapidly evolving customer expectations. This necessitates the organisation of knowledge within an analytical framework that facilitates the identification of patterns, the comparison of results, and the guidance of new research directions.

The absence of a coherent structure hinders the precise identification of the technologies most extensively adopted, the functions prioritised in financial institutions, and the regulatory and operational challenges most prevalent in diverse contexts. ^8^ This absence of integration also hinders the capacity of researchers, regulators, and industry stakeholders to identify good practices, anticipate risks, or formulate effective strategies. This scenario is of particular significance in the context of regulatory affairs, where the discrepancy between technological innovation and institutional response can have a detrimental impact on both the stability of the system and the confidence of its users.

Consequently, a thorough review is imperative to organise the evidence, classify existing contributions, and identify commonalities, thematic gaps, and dominant trends. An integrative approach to knowledge would overcome the current dispersion and consolidate a theoretical and empirical foundation that facilitates future research and actions aimed at strengthening secure, inclusive, and effective digital banking. ^4^

In this sense, the objective of this research is to explore emerging trends in digital banking by identifying adopted technologies, prioritised functionalities, recurring challenges, adoption factors, and recent regulatory approaches. In order to achieve this objective, a series of questions have been devised to guide the analysis and structure the review of available knowledge on digital banking. The purpose of these questions is twofold: firstly, to elucidate the salient aspects of the phenomenon under investigation; and secondly, to facilitate the identification of common patterns in recent literature.

Which technologies have been most widely adopted in recent studies on digital banking?

Which functionalities have been prioritized by financial institutions in the development of digital banking platforms?

What challenges are most frequently reported in the implementation of digital banking solutions?

What factors have been identified as determining factors in user adoption of digital banking?

What regulatory approaches have been proposed or implemented to respond to emerging risks in digital banking?

This study proffers a systematised synthesis of emergent trends in digital banking, integrating technological, functional, regulatory and adoption aspects. The value of the concept lies in its ability to articulate diverse forms of knowledge, thereby facilitating a comprehensive and coherent understanding of the phenomenon. This integration contributes to the advancement of knowledge, facilitating future research, supporting the formulation of public policies, and guiding the design of effective strategies for innovation and sustainable development in the digital financial sector.

## Methodology

The present systematic review is based on the PRISMA 2020 methodology, which is renowned for its rigour in the selection and reporting of scientific studies. This updated guide offers a structured framework that ensures transparency and reproducibility in the synthesis of available evidence. ^9^ The application of the protocol facilitates the systematic identification, evaluation and selection of relevant studies on emerging trends in digital banking, ensuring a thorough and unbiased process. Furthermore, it facilitates the lucid and meticulous presentation of results, thereby enhancing the methodological quality and reliability of the findings. This methodology is thus designed to ensure the integrative analysis is both robust and useful for the scientific community and financial sector stakeholders interested in digital transformation.

### Eligibility criteria

The inclusion criteria considered studies related to digital banking, with a focus on trends, adoption, challenges, technologies, and regulations in this field. A selection of publications in both English and Spanish, covering the period from 2021 to 2025, was made in order to guarantee the timeliness and relevance of the information. The following publications were included in the present study: scientific articles, reviews and technical reports. The inclusion criteria for these publications were as follows: they must provide empirical evidence or conceptual analysis related to the evolution of digital platforms in the financial sector.

The selection process was conducted in three sequential phases of exclusion. The initial phase of the project involved the elimination of indexing errors, including duplicates and documents unrelated to digital banking, through the verification of titles and abstracts. In the second phase of the project, works that were not available in full-text format were excluded due to restrictions imposed by the database. The third phase of the study consisted of a critical review based on the researcher’s predefined criteria of relevance and methodological quality. This entailed the discarding of studies that demonstrated insufficient evidence, methodological limitations, or that failed to meet the analysis objectives. This procedure guaranteed the inclusion of valid and relevant sources for a rigorous analysis of emerging trends in digital banking.

### Sources of information

The Scopus and Web of Science databases were selected on the basis of their broad multidisciplinary coverage and prestige in indexing scientific publications. Scopus encompasses a wide range of subject areas, including the social sciences, technology, and engineering, thus serving as a reliable source for identifying studies on digital banking. It is evident that Web of Science is distinguished by its meticulous approach to the selection of journals and conferences, thereby ensuring the integrity and validity of the sources it encompasses. The two databases under scrutiny both include a significant representation of research in technology and finance, facilitating access to up-to-date and relevant evidence.

The selection of Scopus and Web of Science is driven by the necessity to guarantee the comprehensiveness and rigour of the bibliographic search, which is a fundamental component of a systematic review. Asubiaro, Onaolapo, and Mills ^10^ posit that, despite the presence of regional discrepancies in the scope of these platforms’ coverage, collectively they provide a comprehensive and balanced perspective on global scientific output, particularly with regard to technological innovation and financial services. This combination has been demonstrated to assist in the reduction of geographic and disciplinary biases, thereby facilitating access to a more diverse and high-quality corpus of literature.

The utilisation of Scopus and Web of Science ensures the incorporation of studies employing rigorous methodologies and yielding relevant results for the analysis of emerging trends in digital banking. These databases permit the implementation of specific and contemporary search criteria, thereby optimising the selection of sources that furnish a comprehensive and detailed perspective on the phenomenon under investigation.

### Search strategy

Specific search equations were designed for each database, integrating the inclusion criteria and combining key terms linked to digital banking, technologies, adoption, and regulation. In Scopus, the following equation was applied: TITLE (“digital banking” OR “online banking” OR “mobile banking”) AND TITLE (“trends” OR “adoption” OR “challenges” OR “technology” OR “regulation”) AND PUBYEAR > 2020 AND PUBYEAR < 2026. In Web of Science, an equivalent equation was used that replaces TITLE with TS= and adapts the KEY or AUTHKEY fields as AK=, in addition to using TS= for TITLE-ABS-KEY. These changes ensure compatibility with the syntax of each platform. The formulation was derived directly from the inclusion criteria, ensuring a precise and exhaustive search. A preliminary validation of terms and Boolean operators was performed to optimize the accuracy and relevance of the results obtained.

### Selection process

The selection process was initiated with a filter based on a review of titles and abstracts to identify potentially relevant studies based on the inclusion criteria. The preselected documents were then subjected to a comprehensive review in order to ascertain their eligibility and relevance to the study’s objective. In both stages of the process, inclusion or exclusion decisions were systematically recorded and documented, ensuring transparency and traceability. Specialized tools were employed to manage and organise references, thereby facilitating control and monitoring of the process. The entire procedure was meticulously documented to support the development of the PRISMA flowchart, which visually displays the progress and refinement of the studies included in the systematic review.

**Figure 1.**
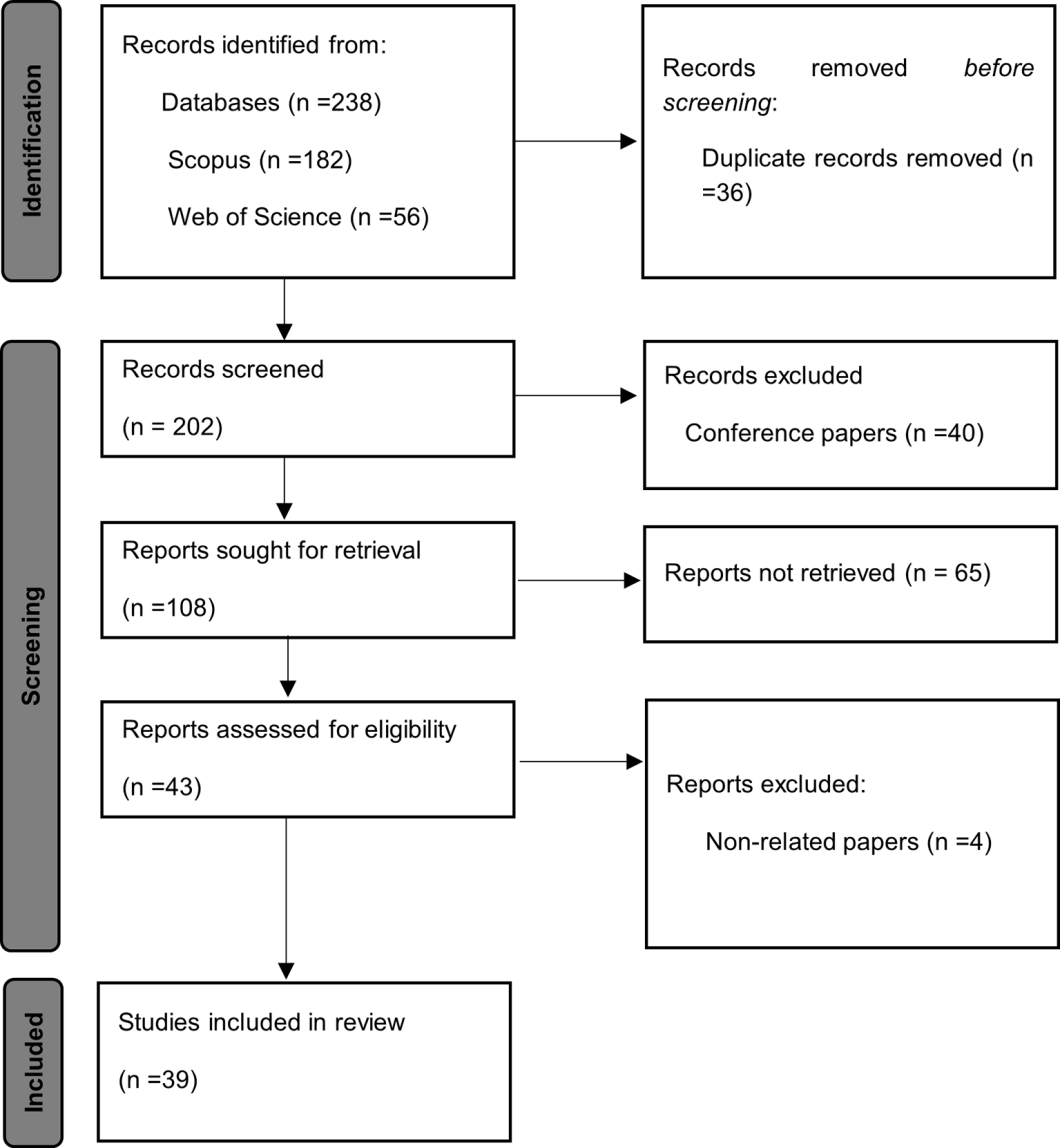
PRISMA flowchart. Prepared by the authors based on Scopus and Web of Science.

The flowchart illustrates the systematic process of identifying, selecting, and excluding studies for the review. The records were initially retrieved from the Scopus and Web of Science databases. Duplicates and records with indexing errors were then removed. The accessibility of the full texts was then assessed, with articles lacking available access being discarded. In conclusion, specific relevance and quality criteria were applied in order to exclude documents that did not meet the study’s requirements. As illustrated in Figure 1, the flowchart delineates the 2020 PRISMA declaration’s recommended protocol, enumerating the number of studies at each stage and thereby demonstrating the transparency and rigour of the selection process.

### Data processing

The data extracted from the selected studies were organised and analysed using Microsoft Excel, allowing for the classification, filtering, and coding of relevant variables for the synthesis of results. Thematic categories related to technologies, functionalities, challenges, adoption, and regulation in digital banking were structured. The use of Excel software enabled a systematic comparison between studies, thus facilitating the identification of emerging patterns and trends. The organised management of the information enabled a rigorous and consistent analysis, thus facilitating the formulation of solid conclusions. Consequently, the implementation of data processing ensured a transparent and replicable process, which is imperative for the methodological quality of the systematic review.

### Risk of bias

The risk of bias was assessed using predefined criteria, including methodological quality, clarity in the presentation of results, and representativeness of the context of the selected studies. This assessment validated the robustness of the synthesis and strengthened the reliability of the conclusions. It was recognised that the exclusivity of the databases and the specific formulation of search terms could generate biases in the selection of evidence. In addition, the original studies’ reporting biases were considered. The bias risk assessment and mitigation process is documented in the flowchart shown in Figure 1, ensuring transparency and methodological rigor in the review.

## Results

The results of this systematic review have been organised according to the research questions, with the analysis being structured around key aspects of digital banking. This organization facilitates the identification of recurring patterns and emerging trends in recent literature, including technologies adopted, functionalities implemented, challenges faced, determining factors in user adoption, and regulatory approaches applied. It is therefore evident that a comprehensive and coherent overview of the current state of knowledge in the field is presented. Table 1 provides a concise overview of the selected studies, which were subjected to a detailed and rigorous analysis.

**Table 1.** Studies included in the research. Prepared by the authors based on Scopus and Web of Science.

Title	Authors
A Model for Analysis of Social Media in Adoption of Mobile Banking	Hamidi & Mohammadi ^27^
An Arab Country’s Digital Shift: A Case Study on Factors Influencing Mobile Banking Adoption in the Arab World	Laradi ^13^
An empirical investigation of the extended Technology Acceptance Model to explain mobile banking adoption	Gokmenoglu & Kaakeh ^17^
Determinants of digital banking adoption in the Kingdom of Saudi Arabia: A technology acceptance model approach	Alnemer ^14^
Examining the dynamics of mobile banking app. Adoption during the COVID-19 pandemic: A digital shift in the crisis	Rahman ^19^
Financial Inclusion of the Elderly: Exploring the Role of Mobile Banking Adoption	Msweli & Mawela ^11^
Impact of CSR activities towards adoption of Mobile Banking	Nguyen & Thao ^28^
Investigating the impact of digital business ecosystem in enhancing Islamic mobile banking adoption through the TOE framework	Abdurrahman ^16^
Islamic mobile banking smart services adoption and use in Jordan	Yaseen ^18^
Mobile banking adoption its antecedents and post-adoption effects: the role of consumers status orientation in an African context	Mefoute Badiang & Nkwei ^29^
Understanding Digital Banking Adoption During Post-Coronavirus Pandemic: An Integration of Technology Readiness and Technology Acceptance Model	Musyaffi ^20^
A STUDY ON ADOPTION OF ARTIFICIAL INTELLIGENCE USE IN MOBILE BANKING	Koyluoglu & Acar ^12^
A Study on the Acceptance of Mobile-Banking Applications in India—Unified Theory of Acceptance and Sustainable Use of Technology Model (UTAUT)	Samartha ^30^
Adoption of Design Thinking, Agile Software Development and Co-creation: A Qualitative Study towards Digital Banking Innovation Success	Indriasari ^31^
Determinants of consumer adoption of Islamic mobile banking services in Indonesia	Febriandika ^32^
Determinants of digital financial exclusion as a barrier to the adoption of mobile banking services in Poland	Solarz & Adamek ^33^
DETERMINANTS Ol CONSUMER ADOPTION Ol ISLAMIC MOBILE BANKING SERVICES IN INDONESIA	Febriandika ^32^
Digital Banking: Challenges, Emerging Technology Trends, and Future Research Agenda	Indriasari ^34^
Evaluation and performance comparison of a model for adoption of biometrics in online banking	Al-Janahi ^35^
Examining Initial Trust in Adoption of Digital Banking Platform: A Personal Innovativeness and Security Perspective	Musyaffi ^36^
Examining the Role of Self-Reliance, Social Domination, Perceived Surveillance, and Customer Support with Respect to the Adoption of Mobile Banking	Asif ^37^
Factors Influencing Mobile Banking Adoption in Cambodia: The Structuring of TAM, DIT, and Trust with TPB	Norng, S. ^38^
How Does Perceived Risk and Trust Affect Mobile Banking Adoption? Empirical Evidence from India	Kumar ^39^
Intelligent Digital Banking Technology and Architecture: A Systematic Literature Review	Indriasari, E., Prabowo, H., Gaol, F. L., & Purwandari, B. ^40^
Intension to Use Mobile Banking: An Integration of Theory of Planned Behaviour (TPB) and Technology Acceptance Model (TAM)	Sasidharan ^41^
ISLAMIC BANK CUSTOMERS’ ADOPTION OF DIGITAL BANKING SERVICES: EXTENDING DIFFUSION THEORY OF INNOVATION	Shaikh ^42^
Online banking adoption in Spanish cities and towns. Finding differences through TAM application	Albort-Morant ^43^
PREDICTING MOBILE BANKING ADOPTION: AN INTEGRATION OF TAM AND TPB WITH TRUST AND PERCEIVED RISK	Obaid ^44^
Prerequisites and perceived information system qualities model for mobile banking adoption among the customers of private commercial banks in myanmar	Tun, P. M. ^45^
Quality factors in technology system capability decision interest in transactions using mobile banking	Suardana ^46^
Role of social media on mobile banking adoption among consumers	Sharma ^47^
Sectoral Comparison of Sustainable Digital Financial Inclusion of Women Workforce with the Mediation of Digital Banking Adoption Intention: An Empirical Analysis	Gupta & Kiran ^48^
Technology adoption of digital banking and women consumers: An empirical investigation	Kaur & Batra ^49^
The Effect of Perceived Usefulness, Reliability, and COVID-19 Pandemic on Digital Banking Effectiveness: Analysis Using Technology Acceptance Model	Ghani ^50^
The Impact of Perceived Risk and Technology Acceptance Model on Gen Z’s Adoption of Digital Banking	Julia ^51^
The Investigation of Preference Attributes of Indonesian Mobile Banking Users to Develop a Strategy for Mobile Banking Adoption	Sebayang ^52^
The role of perceived security and social influence on the usage behavior of digital banking services: An extension of the technology acceptance model	Quynh ^53^
Understanding Consumer Adoption of Mobile Banking: Extending the UTAUT2 Model with Proactive Personality	Hilal ^54^
Unified Theory of Acceptance and Use of Technology (UTAUT) Model: Factors Influencing Mobile Banking Services’ Adoption in China	Mensah ^55^

**Figure 2.**
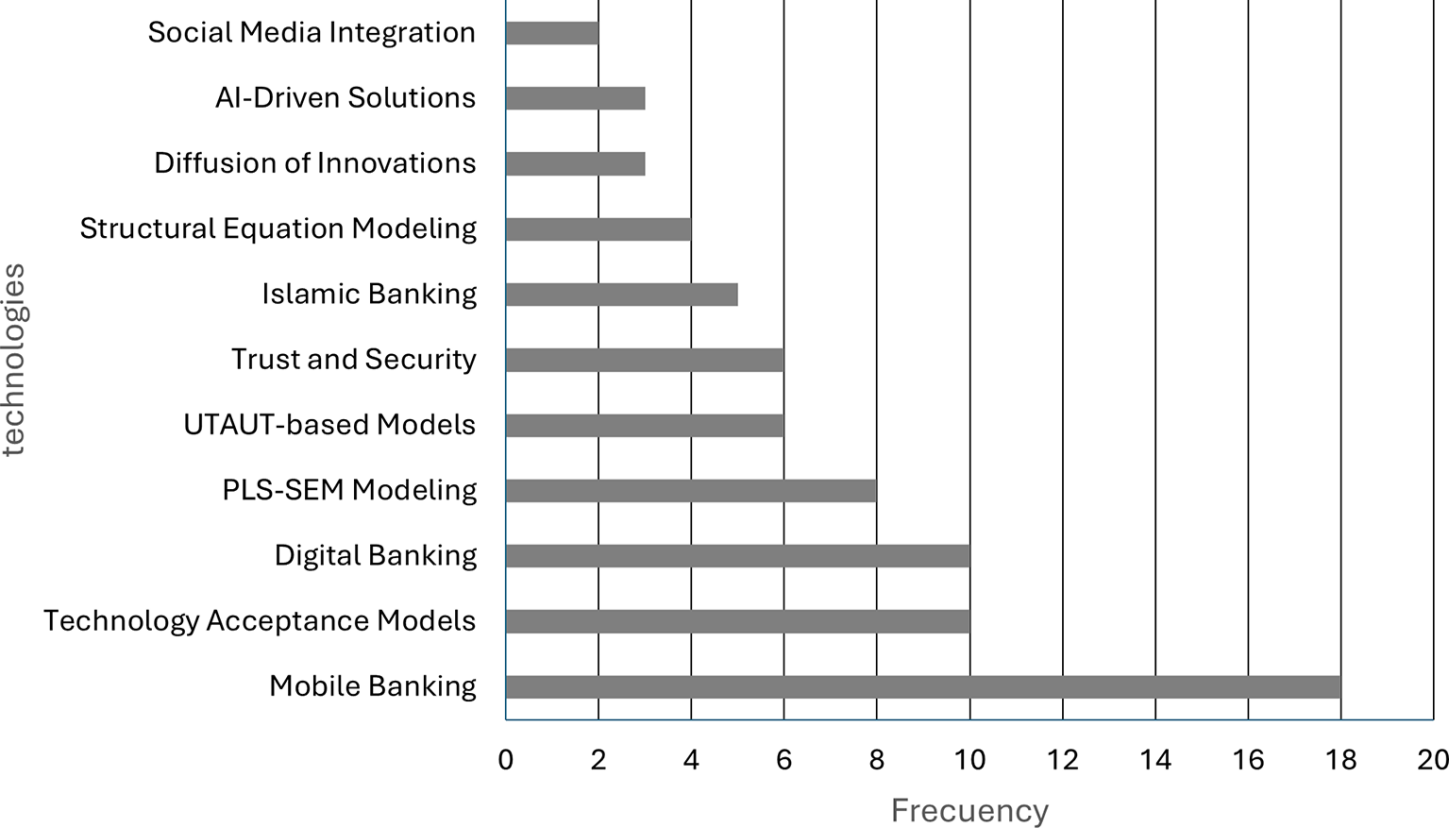
Most widely adopted technologies in digital banking studies. Prepared by the authors based on Scopus and Web of Science.

As illustrated in Figure 2, the predominant technologies employed in digital banking studies are indicated. Mobile banking applications are the subject of the highest number of mentions, with a total of 18. The subsequent themes are technology acceptance models and digital banking approaches, both of which are referenced ten times. PLS-SEM modelling is mentioned on eight occasions. The present analysis indicates that UTAUT-based models and approaches related to trust and security are represented in six studies each. To a lesser extent, there are reports on Islamic banking, SEM modeling, diffusion of innovations, artificial intelligence-based solutions, and social media integration.

**Figure 3.**
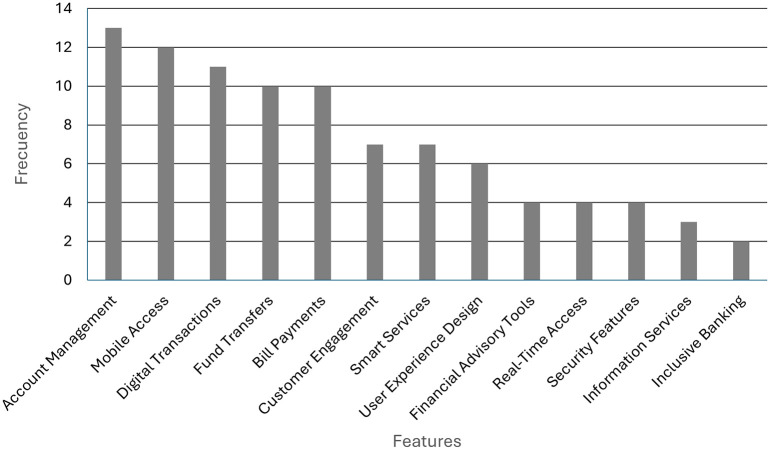
Priority features in digital banking. Prepared by the authors based on Scopus and Web of Science.

As illustrated in Figure 3, the frequency of occurrence of prioritised features in digital banking initiatives documented in the literature is demonstrated. The most frequently mentioned are account management (13), mobile access (12), digital transactions (11), fund transfers (10), and bill payments (10). Subsequent to these are customer engagement, smart services, user-centered design and financial advice. As is evidenced by the paucity of literature on the subject, information services, real-time access, security features and inclusive banking are less frequently mentioned. This distribution enables the identification of the areas most addressed in the functional development of digital solutions in the financial sector.

**Figure 4.**
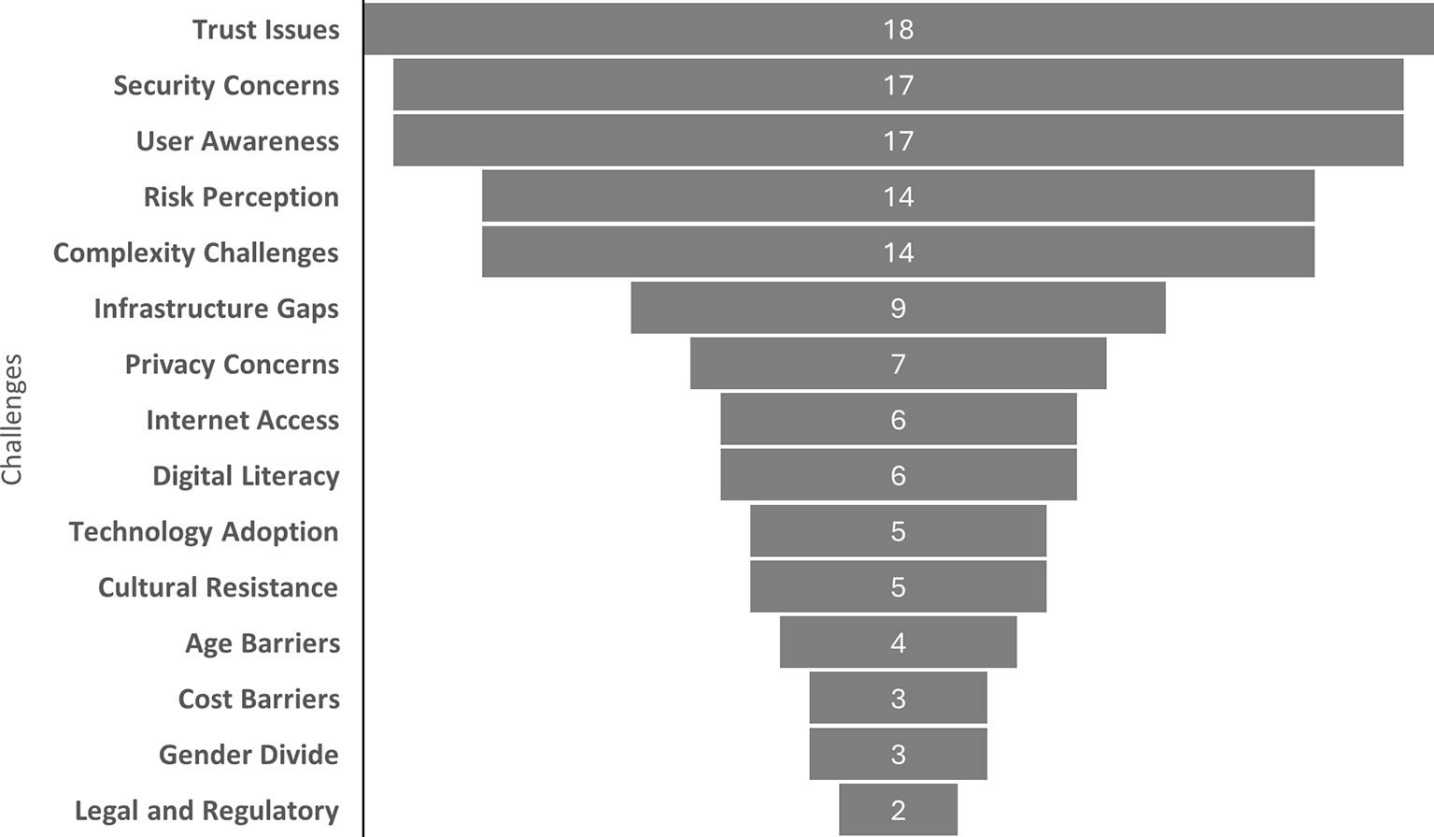
Frequency of common challenges in digital banking. Prepared by the authors based on Scopus and Web of Science.

As demonstrated in Figure 4, the frequency of challenges reported in the domain of digital banking is indicated. The most frequently mentioned issues are trust, security, and user awareness, with 18, 17, and 17 mentions, respectively. Risk perception and complexity appear with 14 mentions each. In addition, infrastructure gaps (9), privacy concerns (7), and internet access limitations (6) have been identified. The presence of gender barriers and legal and regulatory issues is less prevalent, with only two or three mentions.

**Figure 5.**
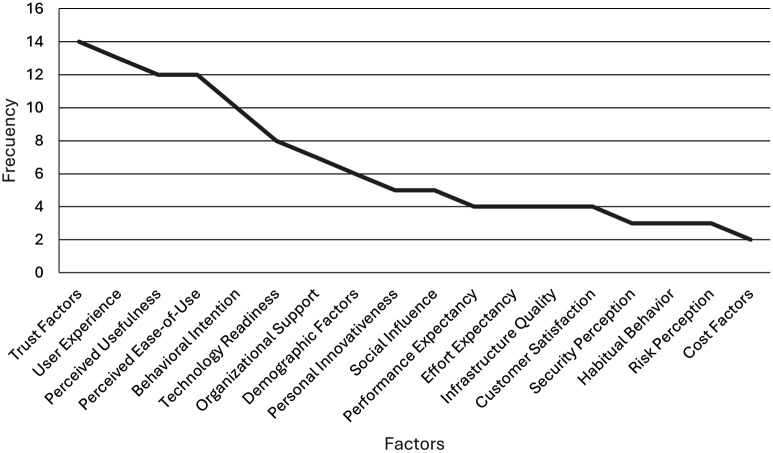
Frequency of determining factors in user adoption. Prepared by the authors based on Scopus and Web of Science.

As illustrated in Figure 5, the frequencies of the categories of determining factors in user adoption of digital banking are demonstrated. Trust and user experience emerged as the most prevalent factors, with 14 and 13 mentions, respectively. It is evident that both the perceived usefulness and the perceived ease of use are discussed at length, with each of these concepts receiving a total of 12 mentions. It is evident that other categories, including behavioural intention, technological readiness, and organisational support, demonstrate a lower frequency of occurrence. The remaining factors have fewer than seven mentions.

**Figure 6.**
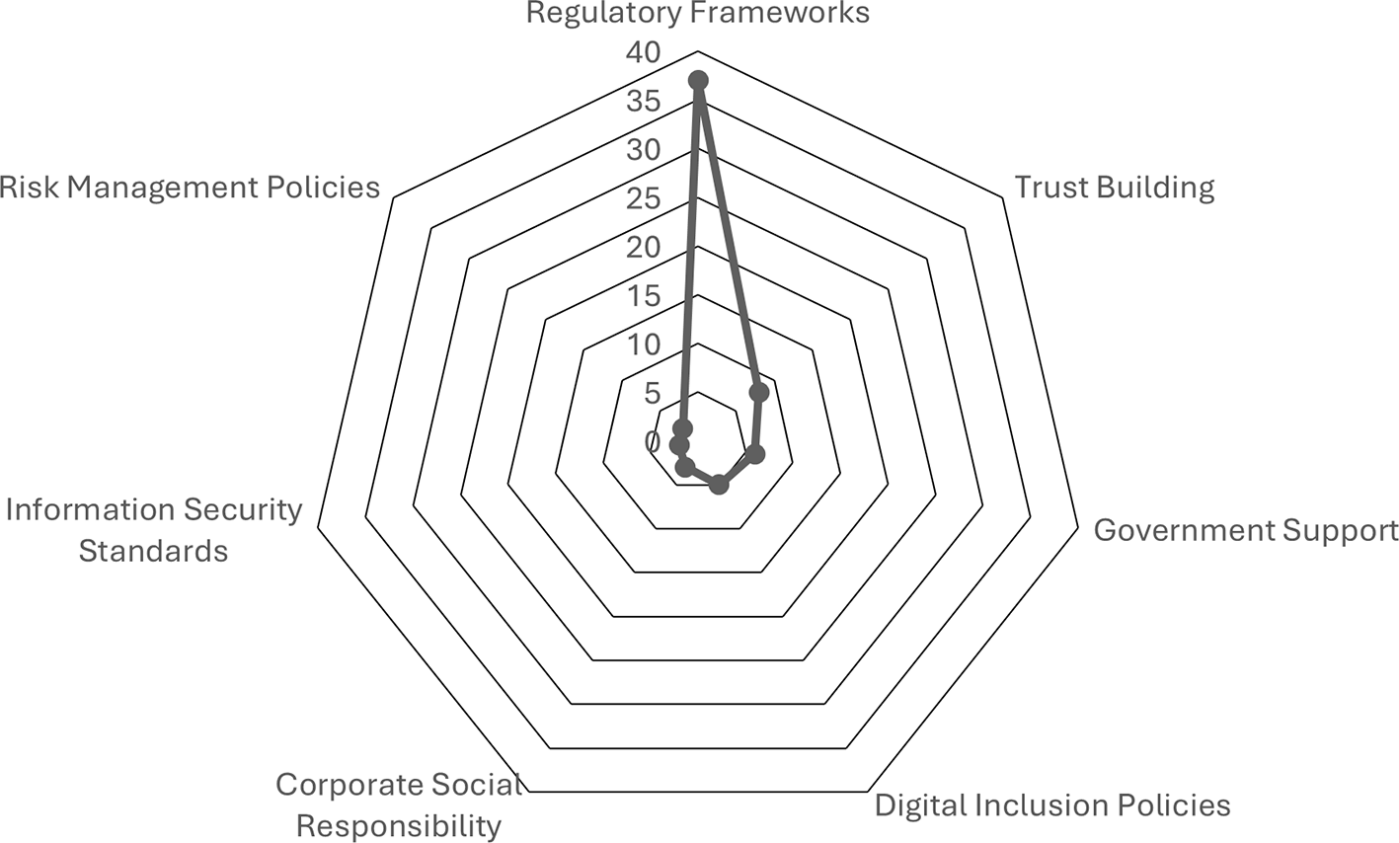
Frequency of regulatory approaches applied. Prepared by the authors based on Scopus and Web of Science.

As illustrated in Figure 6, the frequency of regulatory approaches identified in studies on digital banking is presented. The predominant category is Regulatory Frameworks, with 37 mentions. This is followed by approaches focused on trust building, government support, and digital inclusion policies, with eight, six, and five mentions, respectively. The text also makes mention of Corporate Social Responsibility, Information Security Standards, and Risk Management Policies.

The results were organised according to the research questions and allowed for a structured analysis of emerging trends in digital banking. The review revealed a variety of topics across the studies, covering technologies, functionalities, challenges, adoption factors, and regulatory approaches. This classification facilitated the identification of common patterns, methods, and gaps in the literature. The study also provided a foundation for the comparison of findings and the construction of a conceptual framework. This framework demonstrated the evolution of the digital financial sector and its current priorities from a unified perspective.

## Discussion

The purpose of this section is twofold: firstly, to interpret the findings on emerging trends in digital banking and, secondly, to derive their implications. Initially, the results are compared with those of previous studies in order to establish their position within the existing literature. Subsequently, a conceptual framework is presented, the basis of which is the analysis. The subsequent discussion outlines the theoretical, policy and practical implications of the findings. The study’s limitations, both in its methodological approach and its results, are also noted. Finally, lines of future research are proposed to expand and deepen knowledge in the field of digital banking.

### Analysis of results

The findings indicate that the technologies most frequently studied in the field of digital banking correspond to tools that are characterised by high levels of utilisation and ease of use. Mobile banking is distinguished by its pivotal function in the broader context of financial digitalisation, particularly with regard to the promotion of inclusivity. As posited by Msweli and Mawela, ^11^ the technology in question has been demonstrated to be of use. However, its adoption is said to be limited by issues of trust, a paucity of information, and the complexity of its use. The present study draws upon the analysis of Koyluoglu and Acar ^12^ on the challenges posed by artificial intelligence to perceived usefulness, security, and trust, which are key aspects for achieving sustained user acceptance.

The results indicate a functional orientation in digital banking initiatives, with a focus on basic and operational services that address users’ everyday needs. This phenomenon aligns with the observations reported by Laradi et al., ^13^ who emphasised that perceived usefulness and trust are pivotal factors in the adoption of such services. In a similar vein, Alnemer ^14^ corroborates the notion that ease of use and functionality positively influence user acceptance, while trust functions as a moderating variable. The prioritised functionalities demonstrate an emphasis on operational efficiency, accessibility, and perceived security.

The findings of the study indicate that the primary challenges in the realm of digital banking are attributed to factors such as trust, security, and the absence of user awareness. Risk perception and technological complexity are identified as additional obstacles. The limitations in infrastructure, privacy, and internet access are also evident, while legal, regulatory, and gender barriers are less prevalent. This finding underscores the necessity to bolster trust through actions such as corporate social responsibility, as asserted by Anh and Phuong Thao. ^15^ Similarly, Abdurrahman ^16^ emphasises the pivotal role of the digital ecosystem and the influence of technological, organisational, and environmental factors in the adoption of Islamic mobile banking.

The findings of the study indicate that trust and user experience are the primary factors influencing the adoption of digital banking services. Perceived usefulness and ease of use have been shown to have a significant influence on intention to use. In addition to the aforementioned factors, technological readiness and organisational support have been considered, albeit less frequently. These findings are consistent with previous research highlighting the importance of trust and positive perceptions in fostering adoption, as well as the need to innovate and personalise mobile services to attract users, particularly in emerging markets and Islamic contexts. ^17, 18^

The results of the present study indicate that regulatory frameworks are the most frequent focus in studies on digital banking, thereby highlighting their central role in regulating the sector. Other approaches that have been employed include trust-building, government support, and digital inclusion policies. Although these are less common, they are nevertheless relevant in facilitating the adoption and use of digital services. Similarly, initiatives pertaining to corporate social responsibility, information security standards, and risk management are identified. These regulatory approaches are designed to address the necessity of adapting the regulatory framework in order to cultivate trust in contexts marked by accelerating digitalisation, a need that is particularly pronounced in the post-pandemic era. ^19, 20^

### Comparison of results with other studies

The study results, organised according to the research questions, reveal a wide thematic diversity in digital banking, encompassing emerging technologies, adoption drivers, challenges, and regulatory approaches. This organisation facilitated the identification of patterns and thematic gaps, thereby providing a comprehensive view of the current digital financial sector. In accordance with Munira’s findings, ^21^ the significance of technologies such as artificial intelligence, machine learning, and digital wallets in enhancing operational efficiency, customer experience, and financial inclusion is substantiated.

The findings of both studies underscore the advantages for traditionally underserved sectors, whilst concomitantly highlighting the challenges in cybersecurity and regulatory compliance. However, the present study also incorporates functional and regulatory dimensions, while Munira emphasises technological transformation. As Barru ^22^ have demonstrated, trust, cybersecurity, and financial inclusion are recurring themes in the literature. However, this analysis delves into methodological diversity and less-explored categories, such as organisational support and technological readiness, aspects less highlighted in Barru’s bibliometric analysis.

Moreover, while Barru ^22^ focuses on a specific case (BRImo), this study offers a global perspective, thereby broadening the applicability of the results. In the study conducted by Ahmad, ^23^ the role of digital banking in emerging markets in terms of reducing financial gaps is highlighted. This is accompanied by an emphasis on the social and economic transformation that results from this. The present study corroborates this perspective, incorporating a structured view of technological and regulatory factors that influence inclusion, thus providing a multidimensional understanding of the phenomenon. This perspective is in contrast to the findings of Adebayo et al., ^24^ who conducted a detailed analysis of specific risks in liquidity management due to banking digitalization in the United States.

Whilst the present study does not explore particular financial risks in any depth, it does consider broader regulatory and governance aspects, which may prove of use in future analyses of financial stability in digital environments. The present study finds concordance with the conclusions of Suganya and Yogalakshmi ^25^ with regard to the post-pandemic impact on the acceleration of digital adoption. The findings of both studies concur that the pandemic functioned as a catalyst, emphasising the necessity for regulatory and technological adaptations to ensure the continuity of digital growth. However, this study places greater emphasis on the diversity of regulatory approaches and their significance for trust and digital inclusion.

### Proposed conceptual framework

**Figure 7.**
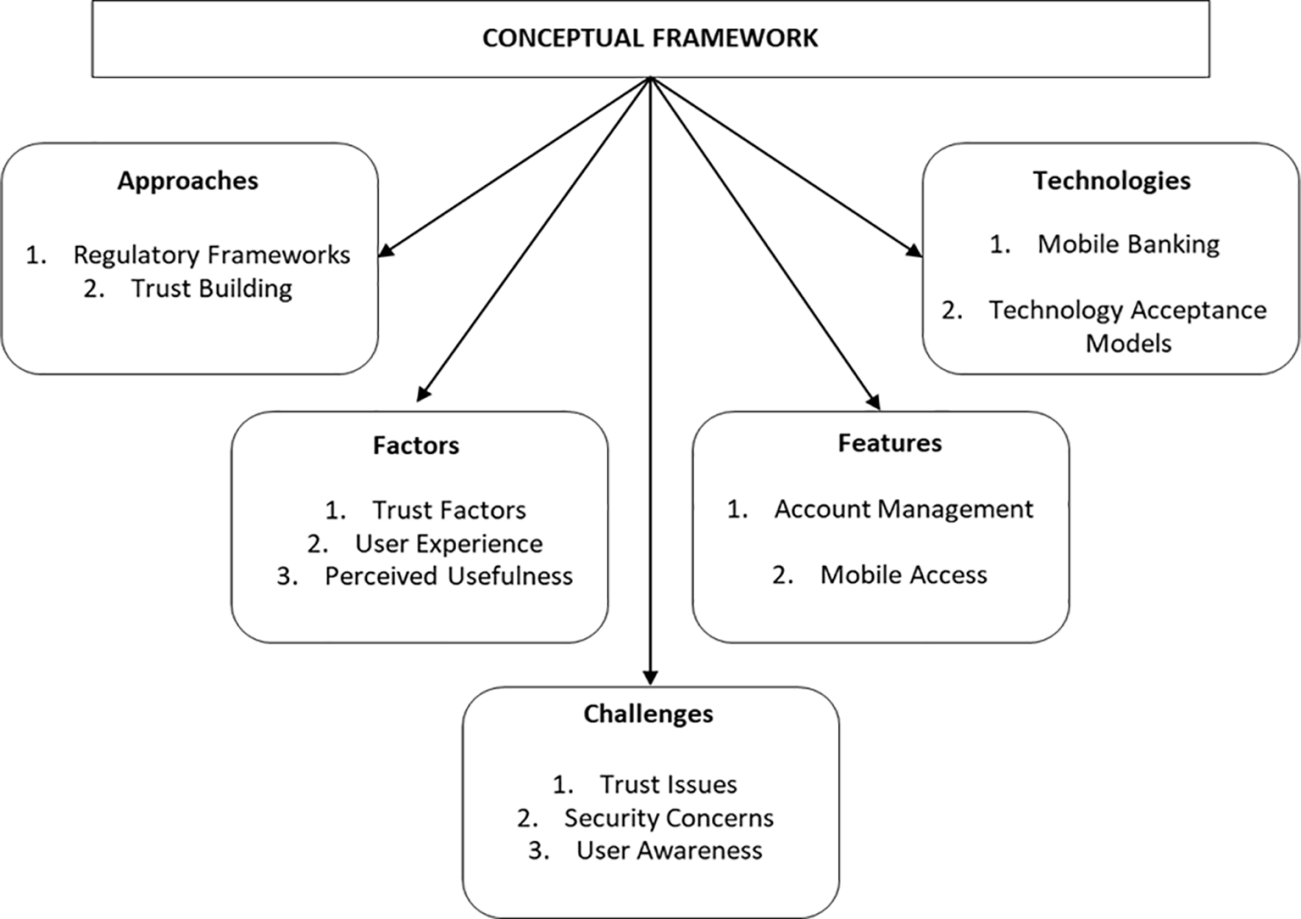
Conceptual framework of trends in digital banking. Prepared by the authors.

The findings of the study yielded an integrated conceptual framework, as illustrated in Figure 7. The model is designed to categorise the primary dimensions of adoption, encompassing the technologies employed, the functionalities prioritised, the challenges encountered, the adoption drivers, and the regulatory approaches adopted. Key interactions among these elements are represented, where technologies enable features that optimize the user experience, challenges affect adoption, and regulatory policies along with organisational support mediate these processes. The framework summarises current trends in digital banking and provides a basis for future analysis and strategic design.

### Implications

The study on emerging trends in digital banking has theoretical, policy, and practical implications, contributing to the understanding and development of the rapidly evolving sector. At the theoretical level, a conceptual framework is proposed that integrates key dimensions: adopted technologies, priority functionalities, challenges, adoption drivers, and regulatory approaches. This model extends and refines previous approaches by incorporating technological, behavioural, regulatory and organisational elements.

The validity of well-established variables such as trust and perceived usefulness is confirmed, whilst simultaneously highlighting the limitations of models that are exclusively focused on technology adoption. Furthermore, under-explored categories, such as technological readiness and institutional support, are incorporated, thus enriching the field and opening new lines of research. From a policy perspective, the results indicate the necessity for comprehensive regulatory policies that consider protection, security, digital inclusion, and trust-building.

It is recommended that regulators devise flexible regulatory frameworks that support technological innovation without neglecting protection against risks such as cyberattacks. It is also emphasised that efforts must be made to promote accessibility and equity in digital services, particularly in emerging markets and across diverse socioeconomic contexts. The articulation between government support, digital inclusion, and security standards is crucial to fostering an ecosystem that drives innovation and protects consumers, thereby promoting the sector’s sustainable development.

In practical terms, financial institutions must adopt comprehensive strategies that consider emerging technologies such as artificial intelligence, blockchain, and digital wallets to improve operational efficiency, personalisation, and accessibility. It is imperative that they cultivate intrinsic competencies to surmount challenges such as cybersecurity, change management, and regulatory compliance, encompassing organisational support policies that facilitate technological adaptation.

It is imperative that developers and user experience designers prioritise security, ease of use, and personalisation, aligning these factors with adoption determinants such as trust and perceived usefulness. The integration of user-centred design methodologies with the implementation of robust security measures is imperative to enhance user acceptance and retention. It is recommended that continuous feedback mechanisms be incorporated in order to adjust features and respond to emerging needs.

When considered as a whole, these implications illustrate the interdependence between the technological, regulatory, and human dimensions that shape digital banking. It is imperative to comprehend these relationships in order to formulate efficacious policies, construct robust theoretical models, and cultivate effective institutional practices that will fortify an inclusive, secure, and adaptable digital financial system. The study provides a solid foundation for formulating comprehensive strategies that respond to current and future challenges, facilitating responsible innovation and financial inclusion in a constantly changing global context.

### Limitations

The study is subject to certain limitations relating to the selection and scope of the database, which focused on indexed literature and academic publications in English. This selection excluded non-indexed sources and documents in other languages, thereby limiting the diversity and representativeness of the perspectives analysed. The inclusion and exclusion criteria, with a focus on studies between 2015 and 2025, restrict the time frame and omit previous developments that could provide historical context or early evolution in digital banking.

The preponderance of quantitative methodologies in the reviewed literature has the potential to skew interpretation towards measurable approaches, thus compromising the value of in-depth qualitative analyses. These limitations, however, result in a reduced generalizability of the results to different contexts or specific, underrepresented financial sectors. Nevertheless, the study maintains its value by offering a systematic and contemporary overview of emerging trends, thereby providing a foundation for future research.

### Lines of future research

It is recommended that future research in the field of digital banking should concentrate on the empirical analysis of emerging technologies that are less frequently present in the extant literature, such as blockchain and advanced artificial intelligence. These technologies have the potential to transform financial processes; however, there is a paucity of detailed studies on their operational impact and user experience. In order to facilitate a comprehensive understanding of the digital ecosystem, it is necessary to explore the adoption of this technology, the technical challenges that are presented by its use, and the benefits that are offered by its implementation.

Furthermore, it is imperative to extend the scope to under-researched regions and geographic segments, particularly emerging markets and communities with limited financial inclusion. These areas demonstrate particular dynamics that may reveal specific barriers or innovative models of digital adoption. The analysis of these contexts will facilitate the design of bespoke policies and strategies that promote equity and accessibility in digital financial services.

The proposed conceptual framework requires validation and enhancement through studies that consider diverse contexts and methodological approaches. The implementation of this model in practical cases is expected to enhance its robustness, thereby facilitating the identification of potential adjustments and supplementary components. In order to obtain a more complete perspective on the phenomenon, it is recommended that mixed methodologies be adopted, incorporating both quantitative and qualitative analyses.

The process of triangulation enables the collection of both general trends and specific aspects, thereby facilitating the generation of robust and applicable conclusions. In summary, research must integrate technological, social and regulatory dimensions from multiple perspectives and contexts if knowledge is to be expanded and the strategic development of digital banking is to be supported in a dynamic and constantly changing global environment.

## Conclusions

This study elucidates the intricacies of the evolution of digital banking, wherein technological, social, and regulatory dimensions converge and exert influence on its development. The variety of approaches and the breadth of topics identified demonstrate that digital transformation in the financial sector is not a simple phenomenon, but rather a dynamic process that requires a comprehensive analysis. It is evident that a comprehensive understanding of digital banking necessitates an examination that extends beyond a purely technical milieu, encompassing its institutional and social ramifications. The adoption and consolidation of digital services are contingent on both technological advancement and the implementation of policies and practices that promote inclusion and reduce risk. The relationship between emerging technologies, the functionalities offered, and challenges related to security and perception creates a scenario in which sector actors must coordinate efforts to balance innovation and protection.

Digital banking is a field that is subject to constant transformation, influenced by diverse socioeconomic contexts and accelerated by external factors, such as the recent pandemic. This reality necessitates that research remain adaptable, expand its scope, and delve into areas that have been under-explored to generate knowledge capable of anticipating and responding to the future demands of the digital financial system.

## Ethics and consent

Ethical approval and consent were not required.

## Data Availability

The data availability statement for this study has been duly registered and archived in the Zenodo open data repository, which is recognized for its commitment to the accessibility and preservation of scientific data. The data and materials supported by this study are publicly available under a Creative Commons Attribution 4.0 International (CC BY 4.0) license and can be accessed at the following DOI link: https://doi.org/10.5281/zenodo.15685817. ^26^
