# Supplementary material for: Emerging trends in digital banking: technologies, usability, regulation, and transformation of the financial sector
Source: F1000Res. 2025 Jul 24;14:727. [Version 1] doi: 10.12688/f1000research.166554.1 (PMC12891957; doi:10.12688/f1000research.166554.1)
Supplement: Peer review history

# Reviewer report: Reviewer response for version 1

**DOI:** 10.5256/f1000research.183559.r427279

**Published:** 2026-02-10

**Authors:** Zaenal Aripin

**Affiliations:** 1 Sangga Buana University, Bandung, West Java, Indonesia

Excellent PRISMA 2020 systematic review analyzing digital banking literature (Scopus & Web of Science, 2021-2025). Identifies 4 key trends: mobile banking dominance (18 studies), core functionalities, trust/security challenges (17-18 mentions each), and regulatory frameworks (37 mentions). Strong visualizations (Figures 1-7) and Zenodo open data.

 **DETAILED ASSESSMENT:**

 **1. Rationale & objectives: APPROVED (minor revision needed)**

 Clear RQs (5 specific questions). **Strength**: Timely post-COVID fintech focus.

 **Weakness**: Introduction overly verbose (~1/3 manuscript length).

 **2. Methods & replicability: APPROVED ✓**

 Excellent execution: dual databases, transparent search equations, complete PRISMA flowchart (Fig 1), Excel thematic analysis, Zenodo DOI data. Fully replicable.

 **3. Statistical analysis: NOT APPLICABLE**

 Appropriate frequency counts (Fig 2-6) for thematic review.

 **4. Conclusions supported: APPROVED ✓**

 Findings directly from data, solid conceptual framework (Fig 7).

 **STRENGTHS:**

 - ✅ Dual database rigor (Scopus+WoS)

 - ✅ Comprehensive visualizations (Fig 2-6)

 - ✅ Zenodo open data transparency

 - ✅ Practical regulatory implications

 - ✅ Global emerging markets coverage

 **MAJOR RESERVATIONS (specific & constructive):**

 1. **Introduction too verbose**: ~33% manuscript = background.

    **Recommendation**: Trim to 15-20% length. Sharpen gap: "Despite 100+ studies, no systematic synthesis exists for 2021-2025 digital banking trends amid post-COVID fintech acceleration."

 2. **Search timing unclear**: 2021-2025 data but Jan 2026 publication.

    **Recommendation**: Add search date: "Literature search conducted Dec 2025" + check for Jan 2026 papers.

 3. **Geographic analysis missing**: Global claims without country breakdown.

    **Recommendation**: Add Table 3: Country distribution from dataset (e.g., "India 25%, Latin America 18%, Europe 15%").

 4. **Risk of bias assessment minimal**: Mentioned but not quantified.

    **Recommendation**: Simple quality table: "80% studies high quality (MMAT criteria)."

 **RECOMMENDED REVISIONS (prioritized):**

 **MAJOR revisions (required for full approval):**

 1. Shorten introduction 30%, sharpen research gap (2 sharp paragraphs)

 2. Clarify search date + update if 2026 papers available

 3. Add geographic distribution table

 **MINOR revisions:**

 1. Risk of bias assessment table

 2. Minor English polishing

 **OVERALL ASSESSMENT**: Excellent systematic review with high practical value for fintech researchers globally. Minor revisions will elevate to outstanding contribution.

Are the rationale for, and objectives of, the Systematic Review clearly stated?

Partly

Is the statistical analysis and its interpretation appropriate?

Not applicable

If this is a Living Systematic Review, is the ‘living’ method appropriate and is the search schedule clearly defined and justified? (‘Living Systematic Review’ or a variation of this term should be included in the title.)

Not applicable

Are sufficient details of the methods and analysis provided to allow replication by others?

Yes

Are the conclusions drawn adequately supported by the results presented in the review?

Yes

Reviewer Expertise:

Knowledge Management, Digital Transformation, Fintech, Strategic Management, Organizational Behavior

I confirm that I have read this submission and believe that I have an appropriate level of expertise to confirm that it is of an acceptable scientific standard.

# Reviewer report: Reviewer response for version 1

**DOI:** 10.5256/f1000research.183559.r427275

**Published:** 2025-11-07

**Authors:** Marshal Iwedi

**Affiliations:** 1 Rivers State University, Nkpolu-Oroworukwo, Nigeria

The paper is well-written, conceptually sound, and methodologically transparent. The structure of the article is in line with standard protocols for systematic reviews, and logical flow interlinks from problem identification to discussion. Application of PRISMA is appropriate and enhances its scientific validity. The article contributes significantly by integrating fragmented literature on digital banking trends and providing an integrated conceptual model. However, several areas need clarification and improvement to make the article scientifically robust and practically useful.

 Key changes in ensuring scientific soundness

 Quantify the PRISMA flow: number of studies identified, screened, excluded, and included.

 Specify the bias assessment tool or criteria used, such as ROBIS or CASP.

 Provide a more analytical synthesis of results, rather than a descriptive summary.

 It should be explained that there is a discrepancy with respect to time frame when comparing 2015–2025 and 2021–2025.

 Enhance discussion by relating findings to existing adoption and regulatory theories.

 Include concise policy examples to illustrate recommendations.

 Improve the presentation of figures and tables by adding numeric context and clearer legends.

Are the rationale for, and objectives of, the Systematic Review clearly stated?

Yes

Is the statistical analysis and its interpretation appropriate?

Yes

If this is a Living Systematic Review, is the ‘living’ method appropriate and is the search schedule clearly defined and justified? (‘Living Systematic Review’ or a variation of this term should be included in the title.)

Yes

Are sufficient details of the methods and analysis provided to allow replication by others?

Yes

Are the conclusions drawn adequately supported by the results presented in the review?

Yes

Reviewer Expertise:

Finance and Digital Banking

I confirm that I have read this submission and believe that I have an appropriate level of expertise to confirm that it is of an acceptable scientific standard, however I have significant reservations, as outlined above.

# Reviewer report: Reviewer response for version 1

**DOI:** 10.5256/f1000research.183559.r405900

**Published:** 2025-09-15

**Authors:** Simran Kaur

**Affiliations:** 1 Babu Banarasi Das University, Lucknow, Uttar Pradesh, India

1. Though the introduction part is well-written, the rationale doesn't seem to be highlighted. Also, a theoretical background related to digital banking can enhance the quality of the paper. 

 2. Various terms used like financial inclusion and consumer behaviour are not properly linked with digital banking.

 3. Research questions need to be re-framed in accordance to the title which speaks of "emerging trends."

 4. Methodology is very well-written and exhaustively each step is explained.

 5. Statistical analysis is represented clearly but interpretation needs to be worked upon. Interpretation can be linked with existing literature to strengthen the analysis part.

 6. Conceptual framework can be incorporated after Introduction part initially. Then later, in Discussion section, it can be discussed whether findings align with the conceptual framework provided.

 7. Implications can be segregated under different headings to make them more presentable.

 8. Conclusion needs to be re-written in line with the title.

Are the rationale for, and objectives of, the Systematic Review clearly stated?

Partly

Is the statistical analysis and its interpretation appropriate?

Partly

If this is a Living Systematic Review, is the ‘living’ method appropriate and is the search schedule clearly defined and justified? (‘Living Systematic Review’ or a variation of this term should be included in the title.)

Not applicable

Are sufficient details of the methods and analysis provided to allow replication by others?

Yes

Are the conclusions drawn adequately supported by the results presented in the review?

Yes

Reviewer Expertise:

Management (Marketing, Finance, IT)

I confirm that I have read this submission and believe that I have an appropriate level of expertise to confirm that it is of an acceptable scientific standard, however I have significant reservations, as outlined above.
